# Decidual-Secreted Factors Alter Invasive Trophoblast Membrane and Secreted Proteins Implying a Role for Decidual Cell Regulation of Placentation

**DOI:** 10.1371/journal.pone.0031418

**Published:** 2012-02-16

**Authors:** Ellen Melaleuca Menkhorst, Natalie Lane, Amy Louise Winship, Priscilla Li, Joanne Yap, Katie Meehan, Adam Rainczuk, Andrew Stephens, Evdokia Dimitriadis

**Affiliations:** Prince Henry's Institute, Clayton, Victoria, Australia; State Key Laboratory of Reproductive Biology, Institute of Zoology - Chinese Academy of Sciences, China

## Abstract

Inadequate or inappropriate implantation and placentation during the establishment of human pregnancy is thought to lead to first trimester miscarriage, placental insufficiency and other obstetric complications. To create the placental blood supply, specialized cells, the ‘extravillous trophoblast’ (EVT) invade through the differentiated uterine endometrium (the decidua) to engraft and remodel uterine spiral arteries. We hypothesized that decidual factors would regulate EVT function by altering the production of EVT membrane and secreted factors. We used a proteomics approach to identify EVT membrane and secreted proteins regulated by decidual cell factors. Human endometrial stromal cells were decidualized in vitro by treatment with estradiol (10^−8^ M), medroxyprogesterone acetate (10^−7^ M) and cAMP (0.5 mM) for 14 days. Conditioned media (CM) was collected on day 2 (non-decidualized CM) and 14 (decidualized CM) of treatment. Isolated primary EVT cultured on Matrigel™ were treated with media control, non-decidualized or decidualized CM for 16 h. EVT CM was fractionated for proteins <30 kDa using size-exclusion affinity nanoparticles (SEAN) before trypsin digestion and HPLC-MS/MS. 43 proteins produced by EVT were identified; 14 not previously known to be expressed in the placenta and 12 which had previously been associated with diseases of pregnancy including preeclampsia. Profilin 1, lysosome associated membrane glycoprotein 1 (LAMP1), dipeptidyl peptidase 1 (DPP1/cathepsin C) and annexin A2 expression by interstitial EVT in vivo was validated by immunhistochemistry. Decidual CM regulation in vitro was validated by western blotting: decidualized CM upregulated profilin 1 in EVT CM and non-decidualized CM upregulated annexin A2 in EVT CM and pro-DPP1 in EVT cell lysate. Here, non-decidualized factors induced protease expression by EVT suggesting that non-decidualized factors may induce a pro-inflammatory cascade. Preeclampsia is a pro-inflammatory condition. Overall, we have demonstrated the potential of a proteomics approach to identify novel proteins expressed by EVT and to uncover the mechanisms leading to disease states.

## Introduction

During the establishment of pregnancy, a human blastocyst must implant into the uterine endometrium to facilitate the formation of a functional placenta. Inadequate or inappropriate implantation and placentation is thought to lead to first trimester miscarriage, placental insufficiency and other obstetric complications [1], [2]. To form a functional placenta, specialized cells, the ‘extravillous trophoblast’ (EVT) engraft and remodel uterine spiral arteries, creating the placental blood supply at the end of the first trimester [3].

The local endometrial environment is likely to play a key role in regulating trophoblast invasion [3]. Prior to implantation and in preparation for pregnancy, stromal cells of the human uterine endometrium undergo ‘decidualization’. Decidualization describes the dramatic differentiation of endometrial stromal cells into decidual cells, which become rounded with altered secretory and ECM expression [4]. Decidualization involves the categorical reprogramming of endometrial stromal cells such that different genes are expressed at different stages of the differentiation process [5]. In women, decidualization begins spontaneously in stromal cells adjacent to spiral arterioles during the mid-secretory phase of the menstrual cycle (5^th^–10^th^ day after luteinizing hormone surge) in response to progesterone and regardless of the presence of a functional blastocyst. If implantation occurs, decidualization intensifies and continues to form the decidua of pregnancy [6].

The decidua is thought to regulate trophoblast invasion and placental formation by regulating expression of locally produced factors including cytokines, integrins and major histocompatibility complex factors [4]. The critical importance of decidualization for the formation of a functional placenta in mice has been unequivocally demonstrated by genetically-modified mouse studies where decidualization defects lead to unregulated trophoblast invasion [7] and/or pregnancy failure [7], [8], [9]. However, unlike women, in mice decidualization is initiated by blastocyst implantation, thus the systems are not analogous. Regardless, recent evidence in women indicates that decidualization is also important in the formation of a functional placenta, with impaired decidualization associated with recurrent miscarriage, preeclampsia (PE) and placenta accreta [10], [11], [12].

The mechanisms by which the decidua might regulate EVT function are not well understood. Many studies have examined the role of leukocytes, particularly uterine natural killer cells in EVT function [13], [14], however the role of decidual cells themselves is understudied. In vitro, conditioned medium from isolated cells of 1^st^ trimester decidual explants impairs invasion of HTR8SV/neo cells (immortalized human EVT [15]) compared to culture media alone [16]. Conditioned media collected from decidual stromal cells isolated from 1^st^ trimester decidua demonstrates concentration dependent invasion of B6Tert cells (immortalized human cytotrophoblast cells [15]) [17]. This invasive capability correlated with the MMP2 activity in these cells [17]. However, in neither of these studies was the control media conditioned with control cells. Recently, Godbole [18] reported enhanced invasion of the choriocarcinoma cell lines JEG-3 and ACH-3P [15] following treatment with conditioned media from primary stromal cells decidualized in vitro compared to the pre-decidualized cells. Further, AC1M88 (fusion of JEG-3 and term trophoblasts [15]) spheroids showed enhanced expansion when cultured on top of primary stromal cells decidualized in vitro compared to non-decidualized cells [19]. These studies strongly indicate that decidualized stromal cells regulate EVT function, however they are not conclusive and importantly, do not utilize primary EVT.

We hypothesised that decidual factors regulate EVT function by altering the production of EVT membrane and secreted proteins. We aimed to identify and validate EVT membrane and secreted proteins regulated by decidualized stromal cells. To our knowledge, this is the first study to investigate how decidual cell secretions regulate trophoblast protein production.

## Materials and Methods

### Tissue collection

This study was approved by the Southern Health Human Research and Ethics Committee (#09317B; #06014C). Written and informed consent was obtained from each patient before surgical intervention.

#### Trophoblast isolation

Normal first trimester placental tissue was collected from healthy women undergoing elective termination of pregnancy (amenorrhea: 7–12 weeks). Tissues were washed in 0.9% saline and transferred to DMEM/F12 Ham media (D8437, Sigma-Aldrich) before transportation to the laboratory for further processing.

**Figure 1:**
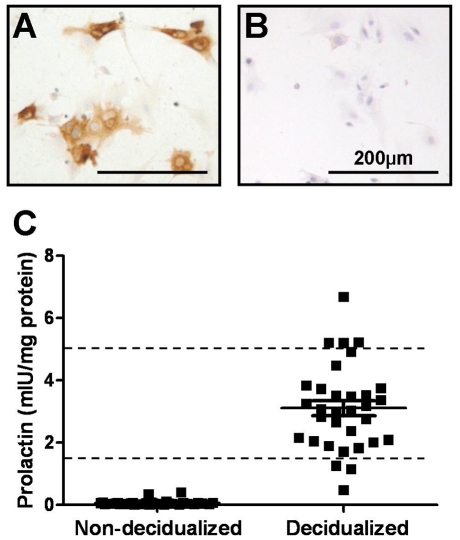
Purity of isolated extravillous trophoblast (EVT) and prolactin secretion by human endometrial stromal cells following decidualization treatment. A. HLAG immunolocalization in isolated EVT. B. Negative (IgG) control for isolated EVT. C. Prolactin secretion by human endometrial stromal cells induced to decidualize in vitro (estrogen [10^−8^ M], medroxyprogesterone acetate [10^−7^ M] and cyclic adenosine monophosphate [0.5 mM]) for 2 (non-decidualized) and 14 (decidualized) days. Conditioned media from decidualized samples containing <1.5 mIU/mg or >5 mIU/mg prolactin (dotted line) were excluded from further studies.

First trimester primary cytotrophoblast were isolated as previously described [20]. The isolated cytotrophoblast were plated on growth factor reduced Matrigel™ (1∶5 dilution in serum-free media; BD Biosciences # 356230) to induce the EVT phenotype [21]. The purity of the isolated EVT was confirmed by immunohistochemistry for HLAG. Briefly, cells were grown on chamber slides coated with Matrigel for 72 h before being fixed in 70% ethanol for 10 min and allowed to air dry. Cells were re-hydrated in dH_2_O for 5 min before exogenous peroxidise blocked by incubation in 3% (v/v) H_2_O_2_ for 15 min. Cells were blocked in 10% normal horse serum, 2% normal human serum for 30 min before primary antibody (HLAG, 0.5 µg/ml, Pharminagen, #557577) was applied in block overnight at 4°C. Cells were washed in Tris buffered saline (TBS) before incubation with a biotinylated horse anti-mouse IgG (1∶200; Vector) secondary antibody for 30 min at RT. This was followed by 30 min incubation with streptavidin-biotin-peroxidase complex ABC (Vector) before HLAG visualized using diaminobenzidine tetrahydrochloride substrate (Dako). Cells were counterstained with Harris haematoxylin (Sigma). This isolation method resulted in 80–95% EVT (HLAG positive cells; Figure 1A&B).

#### Stromal cell isolation

Endometrial biopsies were collected by dilatation and curettage from fertile women scheduled for tubal ligation or undergoing testing for tubal patency during days 8–21 of a normalised 28 day menstrual cycle. Tissues were assessed by a pathologist and had no obvious endometrial pathology. The women had no steroid treatment or other medication for at least 2 months before the collection of tissue.

Human endometrial stromal cells (HESC) were isolated by enzymatic digestion and filtration as described previously [22], resulting in a 97% pure stromal cell culture.

#### In vitro decidualization

HESC (cultured from individual endometrial biopsies) were grown to confluence (maximum time to confluence 5 days) in a T_25_ culture flask (NUNC) in medium (DMEM/F12 [Gibco] supplemented with 10% charcoal stripped Fetal Calf Serum [csFCS; Gibco,], 1% antibiotics and antimycotic [penicillin, streptomycin, amphoceterin B; Gibco] and 1% L-glutamine [Sigma]) in a 5% CO_2_ incubator at 37°C.

Once confluent, the medium was replaced (experimental day [D] 0) with low-serum medium (DMEM/F12+2% csFCS, 1% antibiotics and antimycotic and 1% L-glutamine). Under these conditions cell proliferation is minimal [23], [24], [25]. Cells were decidualized by treatment with 10^−8^ mol/L estradiol 17β (Sigma), 10^−7^ mol/L medroxyprogesterone acetate (MPA; P-0130, Sigma) plus 0.5 mM cyclic adenosine monophosphate (cAMP; Sigma) for 14 days. The media containing treatments was replenished on D2, 4, 7, 9 & 12. Conditioned media (CM) was collected on D2 (non-decidualized CM) and D14 (decidualized CM), centrifuged at 160×*g* to remove any non-adherent cells and stored at −80°C. On D14 cells were lysed and homogenized in ice-cold universal lysis buffer (50 mM Trizma Base [Sigma] pH7.4, 150 mM NaCl, 2 mM EDTA, 2 mM EGTA, 25 mM NaF, 0.2% Triton X-100 [Sigma], 0.3% Nonidet P-40 [Sigma]) containing Protease Inhibitor Mixture Set III (1∶500; Calbiochem) and stored at −80°C. The protein concentration was assayed by commercial kit (BCA, Pierce). HESC prolactin (PRL) secretion was quantitatively measured in conditioned media by ELISA (Bioclone Aust. Pty Ltd, Marrickville, NSW, Australia) and the level of PRL secretion was taken as a measure of decidualization [26]. PRL secretion by non-decidualized HESC was undetectable (Figure 1C). PRL secretion by decidualized HESC was 3.106±1.362 mIU/mg with a range of 0.4714–6.676 mIU/mg (Figure 1C). Decidualized HESC secreting <1.5 and >5 mIU/mg PRL were excluded from further experiments to standardise the level of decidualization across cultures.

### EVT treatment with HESC CM

EVT cultured in a 6 well plate on top of matrigel were treated overnight (16 h) with 500 µl low-serum media or non-decidualized (D2) or decidualized (D14) HESC CM. As a control, each of treatments was also incubated for 16 h in wells coated with Matrigel. These controls were also subjected to the proteomics analysis detailed below to allow exclusion of proteins expressed by the decidua. The EVT/Matrigel CM was centrifuged at 160×*g* to remove cell debris and stored at −80°C. Cell lysates were collected and protein quantified as described above.

#### Conditioned media fractionation

Proteins in the CM with a molecular weight of <30 kDa were fractionated from the remaining media using size-exclusion affinity hydrogel nanoparticles (SEAN) as previously described [27]. Briefly, total protein was precipitated from the CM by incubation in 100% acetone (1 CM : 3 acetone) at −20°C overnight. The protein pellet was resuspended in 10 mM 2-(*N*-morpholino) ethanesulfonic acid, pH 6 before addition of 3 mg SEAN and incubation at RT for 20 min followed by centrifugation to pellet the SEAN. After washes, the fractionated proteins were eluted from the SEAN and concentrated in a centrifugal vacuum concentrator (Eppendorf).

#### Protein identification

Proteins eluted from the SEAN were reduced with 10 mM DTT (Calbiochem) at 56°C for 30 minutes and then thiol groups alkylated with 50 mM iodoacetic acid (Fluka) 30 minutes at RT. Proteins were digested overnight at 37°C with 375 ng trypsin (Worthington). The extracted peptide solution (0.1% formic acid) were then concentrated to approximately 10 µl by centrifugal lyophilisation using a SpeedVac AES 1010 (Savant). Extracted peptides were then injected and fractionated by nanoflow reversed-phase liquid chromatography on a nano LC system (1200 series, Agilent, USA) using a nanoAcquity C18 150 mm×0.15 mm I.D. column (Waters, USA) developed with a linear 60-min gradient with a flow rate of 0.5 µl/min at 45°C from 100% solvent A (0.1% Formic acid in Milli-Q water) to 100% solvent B (0.1% Formic acid, 60% acetonitrile, (Mallinckrodt Baker, Phillipsburg, NJ, USA) 40% Milli-Q water). The nano HPLC was coupled on-line to an LTQ-Orbitrap mass spectrometer equipped with a nanoelectrospray ion source (Thermo Fisher Scientific) for automated MS/MS. Data dependent MS analysis was performed by acquiring one FTMS scan followed by MS2 on the top five most intense ions. Dynamic exclusion was enabled at repeat count 1, exclusion list size 500, exclusion duration 180 s, and exclusion mass width+/−1.5 m/z. Collision induced dissociation was performed by setting the ion isolation width at 2 m/z, normalized collision energy at 35%, activation Q at 0.25 and an activation time at 30 ms.

Spectra were exported in mascot generic file format (.mgf) and analyzed using the Mascot search engine. Standard search parameters included a peptide mass tolerance of 1.5 Da, peptide fragment tolerance of 0.8 Da, peptide charge of +2 or +3 and up to 1 missed cleavage allowed.

Identified proteins were cross-checked between the 6 groups (EVT treated with media alone, EVT treated with ND CM, EVT treated with D CM, media alone, ND CM and D CM). Proteins identified in the media alone control were identified as proteins in the media or expressed by matrigel and excluded from all other groups. Proteins identified in the ND or D CM controls were identified as proteins expressed by HESC and excluded from all other groups. Proteins identified in EVT treated with media alone were identified as proteins expressed by EVT in vitro regardless of treatment and excluded from all other groups. Proteins identified in EVT treated with both ND and D CM were identified as proteins expressed by EVT in response to CM and excluded.

### Validation

#### Immunohistochemistry

Formalin-fixed decidua and placenta sections (5 µm) on poly-l-lysine (Sigma) coated glass slides were dewaxed in histosol (2×10 min) and rehydrated in ethanol. Antigen retrieval in 0.01 M Citrate buffer (pH 6, profilin 1, annexin A2, LAMP1) or 1.25% Trypsin (DPP1) before proteins were co-localized with HLAG using EnVision™ G|2 Doublestain System (Rabbit|Mouse, DAKO, #K5361) as per the manufacturer's instructions. The primary antibodies were incubated at 37°C for 10 min at the following concentrations: profilin 1 0.6 µg/ml (Santa Cruz Biotechnology, sc-137236); annexin A2 0.1 µg/ml (Abcam, #ab41803); DPP1 5 µg/ml (Abcam, #ab49233); LAMP1 3.3 µg/ml (Biolegend, #328602); HLAG 1 µg/ml (Pharmingen, #557577). Negative controls of mouse or rabbit IgG (both DAKO) were applied at the same concentration as the primary antibodies.

#### Western blotting

EVT cell lysates (n = 6) were treated, collected and assayed for total protein as described above. 15 µg total protein (or 1 µg for annexin A2) or conditioned media (28 µl/well, or 10 µl for annexin A2) was resolved on a 1.5 mm 11% SDS/PAGE gel, transferred to Hybond-P PVDF membranes (GE Healthcare) and blocked in 0.1% Tween, 5% skim milk in TBS. Membranes were incubated overnight at 4°C with the primary antibody (all 1∶1000), except GAPDH (HRP-conjugated, Cell Signaling Technology, #3683S, 1∶2000) which was incubated for 1 h at RT. After washes in TBS and 0.1% Tween-20 (BioRad) TBS, HRP-conjugated secondary antibody (profilin 1: sheep anti-mouse, Amersham, 1∶3000, annexin A2 and DPP1, goat anti-rabbit, 1∶5000, secondary antibody not required for GAPDH) was applied, washed again, then the ECL Plus Detection System (GE Healthcare) was applied. Membranes were exposed to autoradiography film (Hyperfilm ECL; GE Healthcare) for between 10 sec and 5 min. Films were scanned and densitometry was performed using Adobe Photoshop. To account for the fact that total protein varied between treatments and a loading control cannot be assigned for conditioned media, the densitometry of the conditioned media was normalized to total cellular protein.

### Statistics

All statistical analyses were performed using GraphPad Prism. Densitometry of western blot data was analysed by paired t-test.

## Results

### Proteomics identified unique proteins in EVT CM in response to treatment with non-decidualized or decidualized CM

Mass spectrometry revealed 43 proteins produced by EVT following the various treatments (Table S1). Of 18 proteins produced by EVT in vitro, 2 were previously unknown to be expressed by cells of the placenta, including EVT (Table S1). Two of the 16 known proteins have previously been associated with pregnancy pathologies (Table S1 [28], [29]).

13 unique proteins were found expressed by EVT in response to treatment with non-decidualized CM, 6 were previously unknown to be expressed by EVT (Table S1) and of the 7 known proteins, 4 are dysregulated in preeclampsia and 1 is associated with endometrial cancer (Table S1 [30], [31], [32], [33], [34]). EVT treated with decidualized CM expressed 12 unique proteins; 6 previously unknown to be expressed by EVT and 5 of the 6 known proteins are dysregulated in either preeclampsia (3), IUGR (1) or in IVF and ICSI pregnancies (1; Table S1 [35], [36], [37], [38], [39]).

Four identified proteins were chosen for validation (Table 1). These proteins were chosen as they are all secreted and have known functions in other cell types which are likely important during EVT invasion into the decidua.

**Table 1:** Proteins chosen for validation by immunohistochemistry and western blot.

Protein	Accession Number	MW (kDa)	Known placenta	Detected by proteomics in	Unique peptides(#)	Sequence coverage(%)
**Profilin 1**	P07737	15	N			
				Media CM	1	11.4
				ND CM	2	20.0
				D CM	4	41.4
**Annexin A2**	P07355	38	Y			
				ND CM	7	25.4
**Dipeptidyl peptidase 1**	P53634	51	N			
				ND CM	1	3.02
**Lysosome-associated membrane glycoprotein 1**	P11279	44	Y	D CM	2	4.8

### Profilin 1

#### Profilin 1 localised to iEVT in decidua basalis

**Figure 2:**
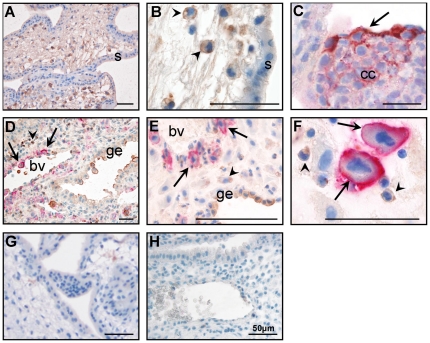
Profilin 1 (brown) and HLAG (pink) co-localization in placental villous (A–C) and decidua basalis (D–F). A. Profilin 1 localized to cells within the stroma of the villous tip, but not the syncytiotrophoblast (s). B. Profilin 1 localized to leukocytes (arrowheads) in the villous tips. C. Profilin 1 and HLAG co-localilzed to extravillous trophoblast (EVT, arrow) in the cell column (cc). D. Profilin 1 localized to the glandular epithelium (ge), and HLAG positive cells (EVT, arrows) surrounding blood vessels (bv) and HLAG negative cells (arrowheads) in the decidua basalis. E. Profilin 1 localized to the ge, EVT (arrows) and HLAG negative cells (arrowheads) in the decidua basalis. F. Profilin 1 and HLAG co-localizaation to EVT (arrows) in the decidua basalis. Profilin 1 was also expressed on leukocytes (HLAG negative cells, arrowheads). G. Negative (IgG) control for placental villous tissue. H. Negative (IgG) for decidua. Scale bar: 50 µm.

Profilin 1 immunolocalized to leukocytes (Figure 2A&B) and EVT in the cell column (Figure 2C) but not to cytotrophoblast or syncytiotrophoblast in the placental villous. Profilin 1 localized to the uterine glandular epithelium (Figure 2D&E), iEVT surrounding remodelled blood vessels (Figure 2D–F) and leukocytes in the decidua (Figure 2E&F).

#### Decidual CM increased profilin 1 secretion

**Figure 3:**
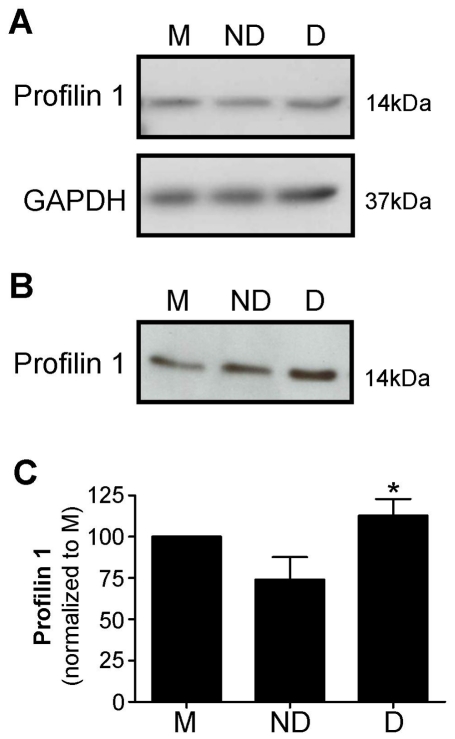
Profilin 1 in extravillous trophoblast cell lysate and conditioned media following treatment with media alone (M), non-decidualized (ND) or decidualized (D) human endometrial stromal cell conditioned media (n = 5). A. Western blot showing profilin 1 and GAPDH loading control in EVT lysates. B. Western blot showing profilin 1 in EVT conditioned media. C. Densitometry of profilin 1 from conditioned media. The ratio of profilin 1:total cellular protein has been normalized to media control. *, significant difference to ND, p<0.05.

Profilin 1 was detected in EVT cell lysates and CM by western blot (Figure 3A&B). Profilin 1 levels in EVT CM were significantly higher in decidualized CM treated EVT compared to non-decidualized CM treated (Figure 3B&C; p<0.05). Profilin 1 was not detected in HESC CM (data not shown). No effect of the week of gestation from which the EVT were isolated was observed.

### Annexin A2

#### Annexin A2 localized to iEVT in decidua basalis

**Figure 4:**
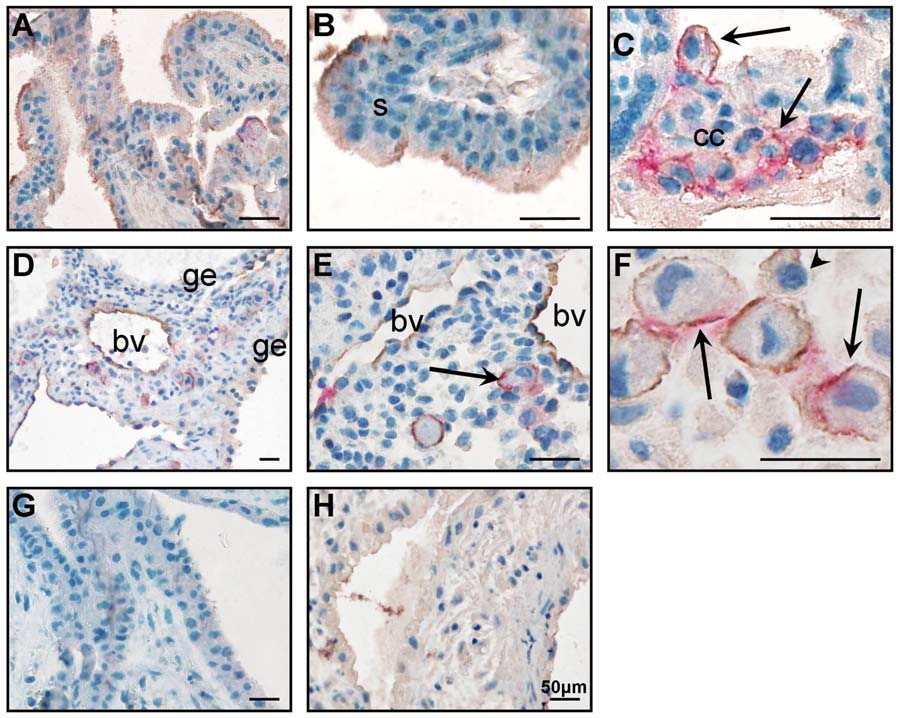
Annexin A2 (brown) and HLAG (pink) co-localization in placental villous (A–C) and decidua basalis (D–F). A. Annexin A2 localization in the placental villous. B. Annexin A2 localized to the syncytiotrophoblast (s). C. Annexin A2 and HLAG co-localilzed to extravillous trophoblast (EVT, arrows) in the cell column (cc). D. Annexin A2 localization in the decidua basalis. E. Annexin A2 localized to endothelial cells of blood vessels (bv) and to iEVT (arrow) in the decidua basalis. F. Annexin A2 and HLAG co-localizaation to EVT (arrows) in the decidua basalis. G. Negative (IgG) control for placental villous. H. Negative (IgG) for decidua. Scale bar: 50 µm.

Annexin A2 localized to the syncytiotrophoblast (Figure 4A&B) and some EVT in the cell column (Figure 4C), but not to cytotrophoblast or leukocytes in the placental villous. Annexin A2 localized to endothelial cells in blood vessels (Figure 4D&E) and iEVT in the decidua (Figure 4E&F).

#### Non-decidualized CM increased annexin A2 secretion

**Figure 5:**
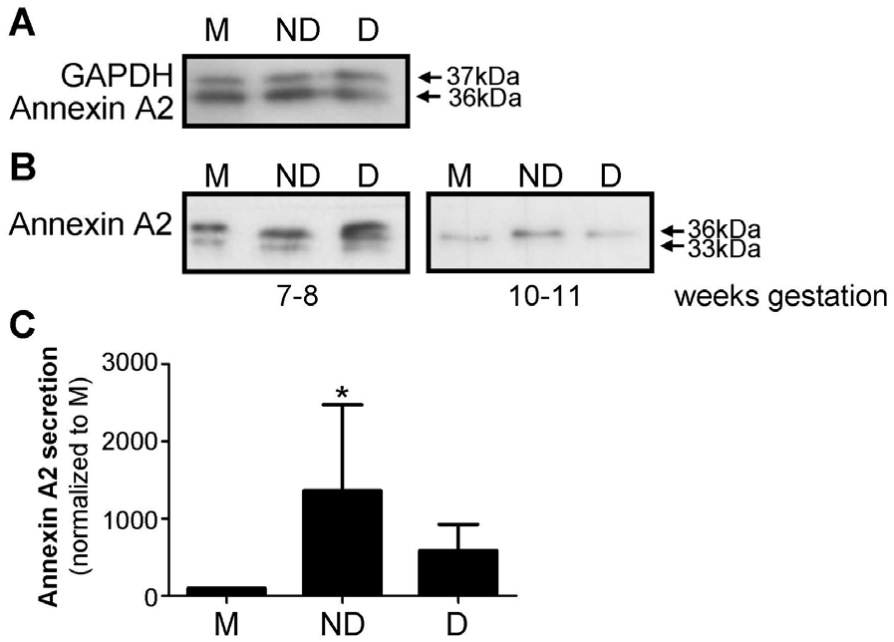
Annexin A2 in extravillous trophoblast cell lysate and conditioned media following treatment with media alone (M), non-decidualized (ND) or decidualized (D) human endometrial stromal cell conditioned media (n = 6). A. Western blot showing annexin A2 and GAPDH loading control in EVT lysates. B. Western blot showing annexin A2 in EVT conditioned media. Shown are representative blots of annexin A2 in EVT isolated from gestational weeks 7–8 (n = 3) and 10–11 (n = 2). Two bands (36 & 33 kDa) for annexin A2 were observed in the conditioned media from EVT isolated from gestational weeks 7–8. C. Densitometry of annexin A2 from conditioned media (all weeks of gestation; n = 6). The ratio of annexin A2:total cellular protein has been normalized to media control. *, signficant difference to M, p<0.05.

Annexin A2 was detected in EVT cell lysates and CM by western blot (Figure 5A&B). HESC CM treatment had no effect on annexin A2 protein levels in EVT cell lysates (Figure 5A). Two bands of annexin A2 (33 and 36 kDa) were observed in CM of EVT isolated from weeks 7 and 8 of gestation but not in CM of EVT isolated from weeks 10 and 11 of gestation (Figure 5B). Secreted annexin A2 protein (36 kDa) in EVT CM was significantly higher (p<0.05) following treatment with non-decidualized CM compared to control (media alone), however the variability between samples was large (n = 6; Figure 5C). Our data suggests the effect of non-decidualized CM was found mainly in EVT isolated from weeks 10 and 11 of gestation (Figure 5B).

### Dipeptidyl peptidase 1 (DPP1)

#### DPP1 localized to iEVT in decidua basalis

**Figure 6:**
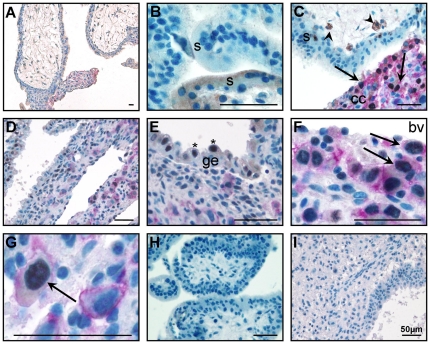
Dipeptidyl peptidase 1 (DPP1; brown) and HLAG (pink) co-localization in placental villous (A–C) and decidua basalis (D–G). A. DPP1 localization in placental villous. B. DPP1 localized to some areas of syncytiotrophoblast (s). C. DPP1 and HLAG co-localilzed to extravillous trophoblast (EVT) in the cell column (cc) (arrows). DPP1 staining was predominantly peri-nuclear in EVT. DPP1 localized to leukocytes in the placental villous stroma (arrowheads). D. DPP1 localization in the decidua. E. DPP1 localized to the glandular epithelium (ge), with peri-nuclear localization observed in some cells (*). F. DPP1 localized to iEVT (arrows) surrounding remodelled blood vessels (bv). G. DPP1 localization in iEVT (arrow). DPP1 localization in iEVT was predominantly peri-nuclear. H. Negative (IgG) control for placental villous. I. Negative (IgG) control for decidua. Scale bar: 50 µm.

DPP1 immunolocalized to some areas of the syncytiotrophoblast (Figure 6A&B), leukocytes (Figure 6B) and to cohorts of EVT in the cell column (Figure 6C) but not to cytotrophoblasts. In the decidua, DPP1 localized to the glandular epithelium (Figure 6D&E) and to iEVT surrounding blood vessels (Figure 6D,F&G). Glandular epithelial and iEVT localization was cytoplasmic, although in EVT the cytoplasmic localization was predominantly around the nucleus.

#### Non-decidualized CM increased proDPP1 production

**Figure 7:**
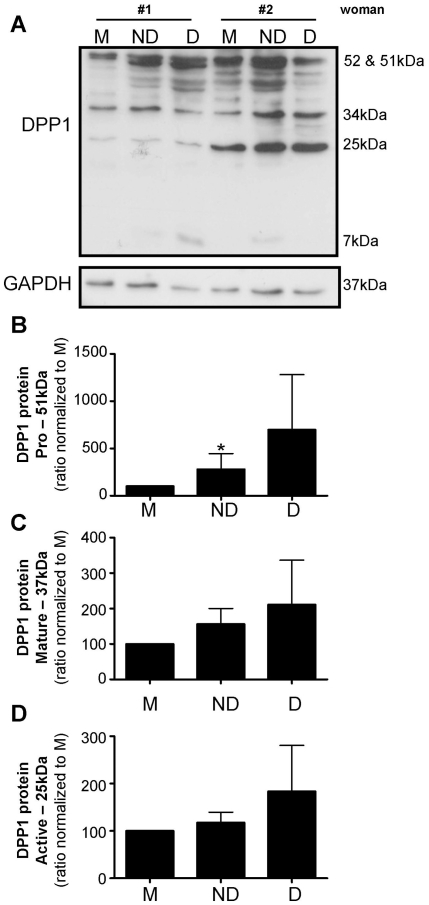
Dipeptidyl peptidase 1 (DPP1) in extravillous trophoblast cell lysate following treatment with media alone (M), non-decidualized (ND) or decidualized (D) human endometrial stromal cell conditioned media (n = 6). A. Western blot showing DPP1 and GAPDH loading control in EVT lysates. B. Densitometry of the 52 & 51 kDa DPP1 band from EVT cell lysates (n = 3). The ratio DPP1∶GAPDH was normalized to media only control. C. Densitometry of the 34 kDa DPP1 band from EVT cell lysates (n = 3). The ratio DPP1∶GAPDH was normalized to media only control. D. Densitometry of the 25 kDa DPP1 band from EVT cell lysates (n = 3). The ratio DPP1∶GAPDH was normalized to media only control. *, significant difference to M, p<0.05.

DPP1 could not be detected in EVT CM by western blotting (data not shown). In EVT cell lysates 5 bands were visible (Figure 7A); 52 kDa and 51 kDa (pro-), 34 kDa (mature), 24 kDa (active) and 7 kDa (mature cleavage) DPP1. By densitometry we found a significant increase in proDPP1 protein in EVT cell lysate following treatment with non-decidualized CM (Figure 7B; p<0.05), but not decidualized CM (p>0.05) compared to media control. There was no effect of HESC CM on mature or active DPP1 (25 kDa; Figure 7C&D). The mature cleavage form of DPP1 (7 kDa) was only observed in EVT cell lysates from two of three women following treatment with HESC CM: in both women the mature cleavage form was present following treatment with non-decidualized CM and in only one woman it was found following treatment with decidualized CM (Figure 7A). All three samples were from EVT isolated from weeks 7–8 of gestation.

### Lysosome associated membrane glycoprotein 1 (LAMP1)

#### LAMP1 localized to iEVT in decidua basalis

**Figure 8:**
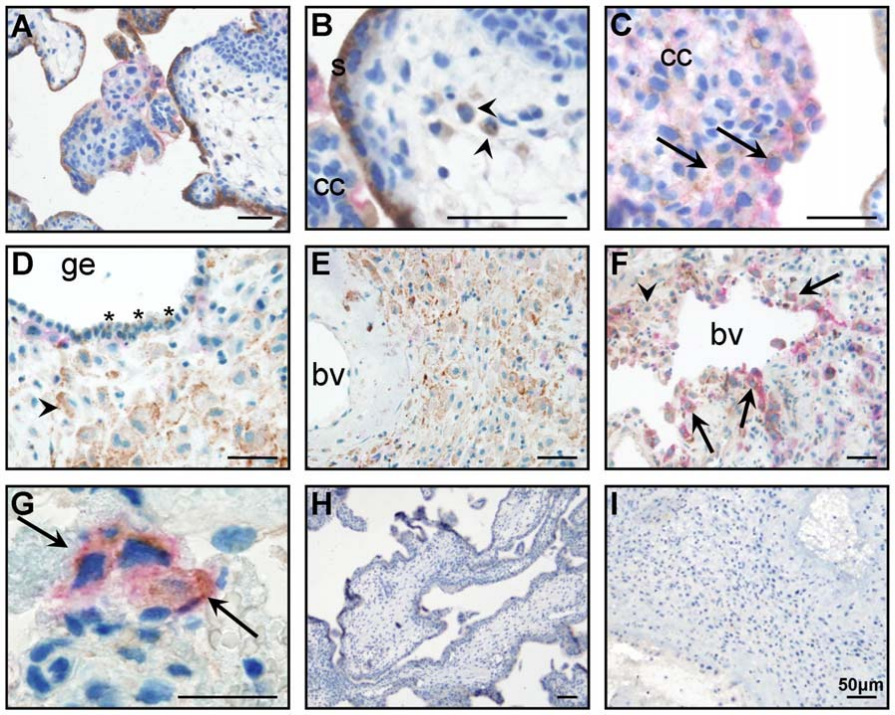
Lysosome associated membrane glycoprotein 1 (LAMP1; brown) and HLAG (pink) co-localization in the placental villous (A–C) and decidua (D–F). A. LAMP1 localization in placental villous. B. LAMP1 localized to the cell column (cc), syncytiotrophoblast (s) and leukocytes in the villous stroma (arrowhead). C. LAMP1 co-localized with HLAG positive cells (arrows) in the cell column (cc). D. The decidual glandular epithelium (ge) was predominantly negative for LAMP1, but LAMP1 immunocolalized to the occasional glandular epithelial cell (*) and to HLAG negative cells in the stroma (arrowhead). E. LAMP1 localized to HLAG negative cells surrounding unremodelled blood vessels (bv). F. LAMP1 localized to EVT (arrows) surrounding remodelled blood vessels (bv). G. LAMP1 and HLAG co-localization (arrows) in the decidua basalis. H. Negative (IgG) control for placental villous. I. Negative (IgG) control for decidua. Scale bar: 50 µm.

LAMP1 immunolocalized to the syncytiotrophoblast (Figure 8A&B) and leukocytes (Figure 8B) in the placental villous and to EVT in the cell column (Figure 8C). In the decidua, LAMP1 localized to occasional cells in the glandular epithelium (Figure 8D), HLAG (EVT marker) negative cells surrounding the glandular epithelium and unremodelled blood vessels (Figure 8D&E) and iEVT surrounding remodelled blood vessels (Figure 8F&G).

LAMP1 could not be detected in EVT CM or cell lysates by western blotting (data not shown).

## Discussion

Here, for the first time, we identified proteins expressed by EVT in response to HESC-secreted factors. Our novel in vitro model identified proteins previously associated with diseases of pregnancy including preeclampsia suggesting our model can identify factors likely to be important in the pathogenesis of these diseases. Our model also identified many proteins not previously known to be expressed by EVTs. We demonstrated that EVT expression of profilin 1, annexin A2 and proDPP1 was altered following treatment with HESC CM, suggesting that the degree of decidualization is critical for decidual-EVT crosstalk during EVT invasion. Further, our data suggests that non-decidualized factors may provoke EVTs to initiate a pro-inflammatory cascade, providing a potential mechanism for the pro-inflammatory state observed in preeclampsia.

Recent studies have suggested that women with preeclampsia have impaired decidualization [12]. Our identified proteins suggest that decidual regulation of EVT protein expression may be associated with the development of preeclampsia, however, future studies are required to validate the regulation of these proteins and their function. By fractionating our sample to identify proteins <30 kDa in size, we aimed to identify functional and regulatory proteins. The main functional groups of proteins identified by our proteomic approach were proteases/enzymes, membrane bound proteins, metal ion binding proteins, and proteins involved in redox regulation. Interestingly, treatment with decidualized CM was associated with an increase in the number of cell membrane proteins expressed by EVT (Table S1) whereas treatment with non-decidualized CM was associated with an increase in the number of proteases expressed by EVT (Table S1). A recent study showed CECAM1 (CD66a) expression by AC1M88 spheriods is enhanced following co-culture with decidualized HESC [19], however to our knowledge this is the first study to suggest that non-decidualized factors regulate protease expression by EVTs.

The EVT proteins identified here included secreted proteins (profilin 1, annexin A2) and cell surface proteins (profilin 1, annexin A2, DPP1, LAMP1). We expected to find cell surface proteins in our CM [40], [41] as our methodology did not exclude cell debris or exosomes given the likely importance of membrane proteins in the regulation of trophoblast invasion. While proteomic analysis of purified EVT plasma membrane would be ideal, we were limited by cell number; isolating purified plasma membrane from primary EVT requires a prohibitively large number of cells.

The decidualization CM used in this study was chosen to reflect the degree of decidualization found in HESC during the 1^st^ trimester; when EVT invade through the decidua and into the top 3^rd^ of the myometrium. Decidual biopsies from weeks 8–10 of gestation secrete 0.3 mIU/mg prolactin in 24 h [42]. Here, prolactin secretion was 3.1 mIU/mg over 48 h, however it should be noted that this was from a purified population of HESC; in a decidual biopsy, less than 50% of the tissue is decidual cells; the tissue includes leukocytes (40% of cells), endothelial cells, smooth muscle and connective tissue. To standardize the degree of decidualization we also excluded under- and over-decidualized CM (<1.5, >5 mIU/mg).

Profilin 1 is an G-actin-binding protein which regulates actin dynamics on the plasma membrane [43] by regulating actin polymerization [44]. Profilin 1 is critical for normal cell proliferation and differentiation and is vital for actin-based cell motility, cytokinesis, neuronal differentiation and regulation of membrane trafficking and nuclear transport [44]. Here, decidualized CM up-regulated profilin 1 levels in EVT CM, suggesting that decidualized CM induced profilin 1 secretion. Profilin 1 secretion by mesenchymal stem cells increases during ionizing radiation induced senescence [45]. In vivo, EVT are non-proliferative; perhaps decidual factors induce senescence in EVT. Certainly, profilin 1 is thought to be a tumor suppressor: it is downregulated in aggressive forms of cancer compared to normal cells [46], [47] and inhibits cell functions required for metastasis including proliferation and migration [46], [48], [49], [50].

EVT are also highly motile and as an actin-binding protein, profilin 1 regulates cell motility, however the exact function of profilin 1 in the regulation of motility is not clear. Although profilin 1 inhibits migration in breast cancer cells, profilin 1 stimulates migration of HUVEC and mesenchymal stem cells [51], [52], [53] and is enriched at the dynamic plasma membranes of migrating or spreading cells [43]. It remains to be determined how the up-regulation of profilin 1 induced by decidualized CM modulates EVT function; the literature currently suggests profilin 1 regulates motility differently between cancerous and normal cells.

Annexin A2 is a calcium-dependent phospholipid binding protein which is present in intracellular, membrane and secreted forms [54]. It has roles in membrane fusion and signal transduction [55] and is over-expressed in a variety of cancers [56], [57]. Secreted and surface annexin A2 interacts with cell matrix and proteases to regulate cell migration and adhesion [57], [58], [59] and it is involved in macrophage activation [60], [61]. Induction or redistribution of annexin A2 has been demonstrated in response to heat shock, hypoxia, cell redox state, mechanical stress and mild osmotic shock [61]. Annexin A2 has previously been identified in the syncytiotrophoblast in vivo [58], [62] and in vitro it is up-regulated by hypoxia in cytotrophoblasts [63] and syncytializing BeWo cells [55], leading to the suggestion that it is part of a defence system against hypoxia [55].

This study is the first to identify annexin A2 in EVT. We found that non-decidualized CM up-regulated secreted/cell surface annexin A2 in EVT isolated from weeks 10 and 11 of gestation. Cell surface expression of annexin A2 is required for invasion and metastasis in cancer [57], [64]. In monocytes and macrophages, surface and soluble annexin A2 (in a tetramer with S100A10) acts as a receptor for plasmin(ogen), which binds then cleaves annexin A2, initiating downstream signalling via multiple pathways including JAK/STAT, MAPK and NF-κβ [60], [65]. This results in the up-regulation of proinflammatory cytokines [60], [65] and thus an inflammatory response [61]. Here we found the cleaved form of annexin A2 (33 kDa) present in CM from EVT isolated from weeks 7 and 8 of gestation but not from weeks 10–12 of gestation, suggesting that plasmin was only active in EVT from weeks 7 and 8 of gestation. Overall our data suggests that surface expression of annexin A2 was normal in EVT from weeks 7 and 8 and likely involved in regulating EVT cell invasion as occurs in uNK cells [66], but that in weeks 10 to 11, annexin A2 surface expression may be an indicator of stress induced by the non-decidualized CM. Certainly, the amount of surface annexin A2 detected by western blot was considerably lower in weeks 10 and 11 compared to 7 and 8. Since plasmin is expressed by eg. uNK cells [66], aberrant surface expression of annexin A2 could lead to abnormal proinflammatory signalling cascades.

DPP1, also known as cathepsin C, is a cysteine protease involved in the activation of pro-inflammatory serine proteases [67], [68], the processing of lysosomal cathepsins and the degradation of intracellular proteins [69]. DPP1 function is best characterised in the immune system: DPP1 deficient mice and human immune cells show reduced NK and T-cell cytotoxic activity [70], [71]. DPP1 activity is highest in lymphocytes with cytolytic potential and myeloid cells [72]. DPP1 activation of granzymes [67] and perforin is thought important in ‘involuntary apoptosis’, whereby target cells are killed without activation of death receptors on the target cell surface [73]. Activation of serine proteases by DPP1 also degrades extracellular matrix, and thus, DPP1 has functions in cell invasion and inflammation [71]. DPP1 transcript has been identified in the placenta [74] however this is the first study to identify DPP1 protein in the placenta. Here we found 5 distinct bands for DPP1: 52 and 51 kDa, the pro-form of DPP1 [75]; 34 kDa, the mature form of DPP1 [76], [77]; 25 kDa, the active form of DPP1 [67] and 7 kDa, the mature cleavage form of DPP1. In this study, proDPP1 was up-regulated by treatment with non-decidualized CM and the mature cleavage form (7 kDa) was found only following treatment with HESC CM. Capthesins are typically localized to lysosomes in the perinuclear region [71] – which we observed here in iEVT and glandular epithelial cells. During cancer development, cathepsins are often translocated to the cell surface or are secreted where they can act as proteases [71]. This has not been demonstrated for DPP1, however it is interesting in light of our proteomics observation that DPP1 was found only in CM following treatment with non-decidualized CM. Certainly, treatment with non-decidualized CM upregulated DPP1 in EVT cell lysates; we were unable to detect DPP1 in EVT CM by western blotting and thus were unable to confirm the regulation of cell surface DPP1 by non-decidualized CM. Overall, our data suggests that the protease activity of DPP1 may be enhanced by non-decidualized CM.

LAMP1, also known as CD107a, was recently identified in EVT [78] where its expression correlated with the ability of EVT to resist infection from *Listeria monocytogenes* [78], suggesting that EVT may have a bactericidal phenotype. Certainly, NK cells which express LAMP1 are ‘degranulating’, ie. they are secreting antimicrobial cytotoxic molecules [79], [80]. Our co-localization data showed HLAG negative, LAMP1 positive cells in the decidua, particularly surrounding un-remodelled blood vessels and the glandular epithelium. These cells did not co-localize with CD45 or CK7 (data not shown), suggesting that these cells are not leukocytes or HLAG negative trophoblast cells. It is interesting to speculate that these cells may be decidual cells involved in the removal of smooth muscle from around spiral arteries.

Here, using a novel in vitro and proteomics approach we have identified a number of proteins which were previously unknown to be expressed by EVT. This approach also identified a number proteins previously associated with diseases of pregnancy, in particular preeclampsia, suggesting that this approach can identify proteins which may have diagnostic or therapeutic potential for targeting these diseases. Previous studies have demonstrated that proteins which are produced at the fetal-maternal interface and regulate trophoblast invasion can be detected and in fact are dysregulated in maternal serum prior to disease onset [81], [82]. Profilin 1, annexin A2 and DPP1 were identified for the first time in EVT and immunolocalized to interstitial EVT in 1^st^ trimester decidua and placental villous (n = 5/6 per group). Further, the expression of profilin 1, annexin A2 and proDPP1 was regulated by HESC CM, indicating that the degree of decidualization may be important for EVT protein production. Interestingly, the two proteins identified as being up-regulated by non-decidualized CM (annexin A2 and DPP1) are both associated with the activation of serine proteases and both induce a pro-inflammatory response, indicating that impaired decidualization may cause excess inflammation during iEVT invasion.

Overall, we have demonstrated that our unique culture method coupled with a proteomics approach has significant potential to identify novel proteins expressed by EVT and to uncover the mechanisms leading to disease states.

## Supporting Information

Table S1 **Proteins identified in EVT conditioned media.**
(DOC)
