# Supplementary material for: Decidual-Secreted Factors Alter Invasive Trophoblast Membrane and Secreted Proteins Implying a Role for Decidual Cell Regulation of Placentation
Source: PLoS One. 2012 Feb 16;7(2):e31418. doi: 10.1371/journal.pone.0031418 (PMC3281063; doi:10.1371/journal.pone.0031418)
Supplement: Table S1 — Proteins identified in EVT conditioned media. (DOC) [file pone.0031418.s001.doc]

Table S1. Proteins identified in EVT conditioned media.

| **Protein** | **Accession Number** | **MW**  **(kDa)** | **Unique Peptides**  **(#)** | **Sequence coverage**  **(%)** | **Function** | **Known placenta** | **Pathology** |
| --- | --- | --- | --- | --- | --- | --- | --- |
| **Media** |  |  |  |  |  |  |  |
| Hemoglobin subunit β | P68871 | 15 | 5 | 49.0 | Iron-transport protein | Y |  |
| Lysozyme C | P61626 | 16 | 5 | 45.9 | Glycolytic enzyme | Y |  |
| CXCL3 | P19876 | 11 | 4 | 39.3 | Chemokine | Y |  |
| Histone H4 | P62805 | 24 | 4 | 21.2 | Histone |  |  |
| CXCL1 | P09341 | 11 | 3 | 38.3 | Chemokine | Y |  |
| Actin, cytoplasmic 2 | P63261 | 41 | 3 | 8.3 | Cytoskeletal protein | Y |  |
| Pregnancy-specific β-1 glycoprotein 3 | Q16557 | 47 | 3 | 8.9 | Membrane protein; CD66f | Y |  |
| Histone H2A type 1-B/E | P04908 | 14 | 2 | 43.8 | Histone |  |  |
| Histone H2A type 2-C | Q16777 | 13 | 2 | 29.2 | Histone |  |  |
| Histone H2A.x (H2AFX) | P16104 | 15 | 2 | 26.6 | Histone |  |  |
| CXCL10 | P02778 | 10 | 2 | 26.5 | Chemokine | Y |  |
| CXCL5 | P42830 | 11 | 2 | 25.4 | Chemokine | Y | HELLP [29] |
| GAPDH | P04409 | 36 | 2 | 9.3 | Glycolytic enzyme | Y |  |
| Plasminogen activator inhibitor 1 | P05121 | 45 | 2 | 8.2 | Serine protease inhibitor | Y | PE [28] |
| *Metallothionein 1X* | *P80297* | *6* | *1* | *19.7* | *Copper-zinc sequester; urea-binding protein* | *N* |  |
| Interleukin 8 | P10145 | 11 | 1 | 16.2 | Chemokine | Y |  |
| ***Profilin 1*** | ***P07737*** | ***15*** | ***1*** | ***11.4*** | ***Actin binding protein*** | ***N*** |  |
| Brain acid soluble protein 1 | P80723 | 22 | 1 | 4.4 | Membrane protein | Y |  |
| **Non-decidualized CM** |  |  |  |  |  |  |  |
| **Annexin A2** | **P07355** | **38** | **7** | **25.4** | **Cell motility** | **Y** |  |
| *Fibrinogen α chain* | *P02671* | *94* | *3* | *4.3* | *Soluble plasma glycoprotein* | *N* | *PE * [30]* |
| E-FABP | Q01469 | 15 | 2 | 17.0 | Fatty acid binding protein | Y | EC [32] |
| Apolipoprotein A1 | P02647 | 30 | 2 | 15.0 | Lipid metabolism | Y | PE [31] |
| *Calmodulin-like protein 5* | *Q9NZT1* | *15* | *2* | *9.6* | *Calcium binding protein* | *N* |  |
| Caspase 14 | P31944 | 27 | 2 | 8.7 | Protease | Y |  |
| *Cytochrome c oxidase subunit 7A2 mitochondrial* | *P14406* | *9* | *1* | *15.7* | *Enzyme* | *N* |  |
| Thioredoxin | P10599 | 11 | 1 | 12.4 | Antioxidant | Y | PE [33] |
| *Calmodulin-like protein 3* | *P27482* | *16* | *1* | *8.1* | *Calcium-binding protein* | *N* |  |
| Peroxiredoxin-2 | P32119 | 21 | 1 | 5.6 | *Redox regulation* | Y | PE [34] |
| *Retroviral-like aspartic protease 1* | *Q53RT3* | *36* | *1* | *3.8* | *Protease* | *N* |  |
| ***Dipeptidyl peptidase 1*** | ***P53634*** | ***51*** | ***1*** | ***3.0*** | ***Cysteine protease*** | ***N*** |  |
| Junction Plakoglobin | P14923 | 62 | 1 | 2.1 | Cytoplasmic junction protein | Y |  |
| **Decidualized CM** |  |  |  |  |  |  |  |
| CD59 | P13987 | 14 | 3 | 23.4 | Membrane protein | Y | PE [37] |
| **Lysosome-associated membrane glycoprotein 1** | **P11279** | **44** | **2** | **4.8** | **Membrane glycoprotein**  **CD107a** | **Y** |  |
| *Ig kappa chain C* | *P01834* | *11* | *1* | *18.9* | *Antibody peptide* | *N* |  |
| *Small proline-rich protein 2E* | *P25531* | *7* | *1* | *12.5* | *Envelope protein* | *N* |  |
| *SH3 domain-binding glutamic acid-rich-like protein 3* | *Q9H299* | *10* | *1* | *10.8* | *Redox regulation* | *N* |  |
| Ferritin Light Chain | P02792 | 20 | 1 | 8.6 | Iron storage | Y | IVF, ICSI [39] |
| *40S ribosomal protein S19* | *P39019* | *16* | *1* | *6.9* | *Ribosomal* | *N* |  |
| *Translocon-associated protein subunit alpha* | *P43307* | *32* | *1* | *5.2* | *Endoplasmic reticulum membrane receptor* | *N* |  |
| Annexin A5 | P08758 | 35 | 1 | 5.0 | Cell motility | Y | IUGR [38] |
| Insulin growth factor binding protein 1 | P08833 | 27 | 1 | 4.3 | Insulin binding protein | Y | PE [35] |
| *Transmembrane glycoprotein NMB* | *Q14956* | *63* | *1* | *3.2* | *Transmembrane glycoprotein* | *N* |  |
| Collagen α-1(XVIII) chain | P39060 | 178 | 1 | 1.0 | Extracellular matrix | Y | PE [36] |

*Italics,* proteins not previously identified in the placenta; **bold,** proteins validated in this study.
